# Partnerships in a Global Mental Health Research Programme—the Example of PRIME

**DOI:** 10.1007/s40609-018-0128-6

**Published:** 2018-10-12

**Authors:** Erica Breuer, Charlotte Hanlon, Arvin Bhana, Dan Chisholm, Mary De Silva, Abebaw Fekadu, Simone Honikman, Mark Jordans, Tasneem Kathree, Fred Kigozi, Nagendra P. Luitel, Maggie Marx, Girmay Medhin, Vaibhav Murhar, Sheila Ndyanabangi, Vikram Patel, Inge Petersen, Martin Prince, Shoba Raja, Sujit D. Rathod, Rahul Shidhaye, Joshua Ssebunnya, Graham Thornicroft, Mark Tomlinson, Tedla Wolde-Giorgis, Crick Lund

**Affiliations:** 1 Alan J. Flisher Centre for Public Mental Health, University of Cape Town, Cape Town, South Africa; 2 Centre for Global Mental Health, Health Service and Population Research Department, Institute of Psychiatry, Psychology and Neuroscience, Kings College London, London, UK; 3 Department of Psychiatry, School of Medicine, College of Health Sciences, Addis Ababa University, Addis Ababa, Ethiopia; 4 South African Medical Research Council, Durban, South Africa; 5 Centre for Rural Health, University of KwaZulu-Natal, Durban, South Africa; 6 Regional Office for Europe, World Health Organisation, Copenhagen, Denmark; 7 Wellcome Trust, London, UK; 8 CDT-Africa, College of Health Sciences, Addis Ababa University, Addis Ababa, Ethiopia; 9 Brighton and Sussex Medical School, Brighton, UK; 10 Perinatal Mental Health Project, Alan J Flisher Centre for Public Mental Health, University of Cape Town, Cape Town, South Africa; 11 School of Medicine, Makerere University, Kampala, Uganda; 12 Transcultural Psychosocial Organization (TPO) Nepal, Kathmandu, Nepal; 13 Aklilu Lemma Institute of Pathobiology, Addis Ababa University, Addis Ababa, Ethiopia; 14 Sangath, Porvorim, India; 15 Ministry of Health, Kampala, Uganda; 16 Harvard Medical School, Boston, USA; 17 BasicNeeds, Bengaluru, India; 18 Department of Population Health, London School of Hygiene and Tropical Medicine, London, UK; 19 Public Health Foundation of India, Bhopal, India; 20 Butabika National Mental Hospital, Kampala, Uganda; 21 Alan J. Flisher Centre for Public Mental Health, Stellenbosch University, Stellenbosch, South Africa; 22 Federal Ministry of Health, Addis Ababa, Ethiopia

**Keywords:** Global mental health, Global health, Partnerships, Low- and middle-income countries

## Abstract

Collaborative research partnerships are necessary to answer key questions in global mental health, to share expertise, access funding and influence policy. However, partnerships between low- and middle-income countries (LMIC) and high-income countries have often been inequitable with the provision of technical knowledge flowing unilaterally from high to lower income countries. We present the experience of the Programme for Improving Mental Health Care (PRIME), a LMIC-led partnership which provides research evidence for the development, implementation and scaling up of integrated district mental healthcare plans in Ethiopia, India, Nepal, South Africa and Uganda. We use Tuckman’s first four stages of forming, storming, norming and performing to reflect on the history, formation and challenges of the PRIME Consortium. We show how this resulted in successful partnerships in relation to management, research, research uptake and capacity building and reflect on the key lessons for future partnerships.

## Background

Global mental health encompasses the study, research and practice of equitably improving mental health for all people (Patel and Prince 2010). Because this field is global and multidisciplinary (Koplan et al. 2009) collaborative research partnerships are required to answer key questions such as how to prioritise mental health at policy level, strengthen mental health service delivery by integrating it into the healthcare system and increase the population demand for services.

Research partnerships provide an opportunity to combine the skill sets of clinicians, researchers, policy makers, service users and service providers across diverse settings. This ensures a good understanding of each country context, including cultures, languages, concepts of mental illness and health systems, resulting in contextualised evidence and enhanced local buy-in and uptake. Partnerships also influence the ability to increase access to funding, credibility and power to affect policy and practice (Afsana et al. 2009). Partnerships can be based on informal arrangements between organisations, formal memoranda of understanding or legally binding contracts (Mirzoev et al. 2012).

Because of the focus on equity in global mental health, countries who currently have the least access to mental health treatment, generally low- and middle-income countries (LMIC), should be at forefront of this work and, therefore, at the heart of global mental health partnerships. However, historically, funding for collaborative research partnerships in global health were obtained from development or research funders in HIC, with programmes led by institutions and researchers in HIC. Researchers in HIC would drive the research questions, allocation of resources and the leadership of the partnership. As a result, the global health research relationships between HIC and LMIC have been criticised for being “semi-colonial” and with inequitable relationships between HIC and LMIC (Costello and Zumla 2000; Tomlinson et al. 2014). Even the more equitable models of partnerships, such as those where research is led by researchers in LMIC and provided with technical support by researchers in HIC (Costello and Zumla 2000), may still assume that technical expertise resides in HIC.

In this paper, we argue that mutually beneficial global health partnerships can and should be based on a synergistic flow of complementary skills and experience. Indeed, in the field of global mental health, where contextual factors are key to understanding the symptoms and expression of mental illness and providing high-quality care in scarce-resource contexts, LMIC partners may have more expertise than HIC partners.

In order to consider how partnerships can work equitably in global mental health, we share our experience of the Programme for Improving Mental Health Care (PRIME), a research programme consortium funded initially for six years by the UK’s Department for International Development (DFID). PRIME aimed to provide research evidence on the integration of mental health into primary and maternal health services in Ethiopia, India, Nepal, South Africa and Uganda.

**Fig. 1:**
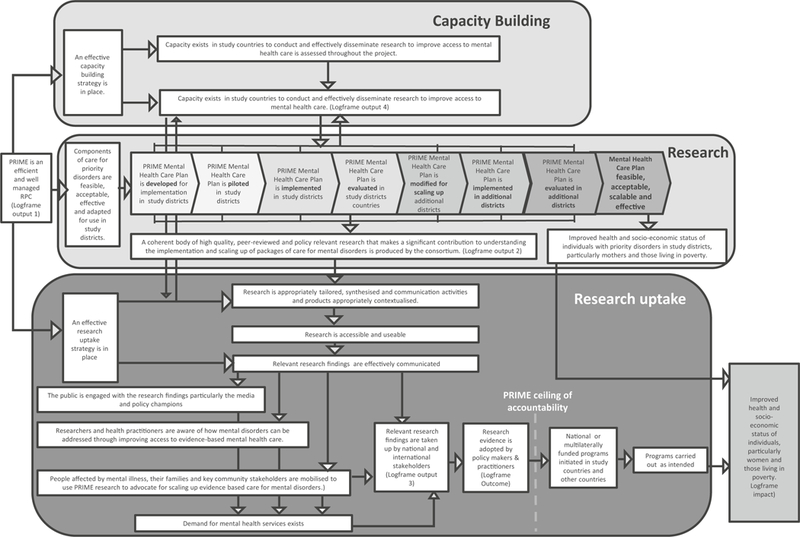
PRIME consortium theory of change

In this paper, we reflect on the history, formation, challenges and achievements of the PRIME partnership across the partner organisations. In order to capture the development of the partnership over time we structured our paper using the first four stages of Tuckman’s model of team development (Tuckman 1965; Tuckman and Jensen 1977). This is a model which describes the formation of teams in relation to the group structure and their orientation to the tasks they need to perform. It has been used widely to understand the development of teams across sectors including the development of public health partnerships (McMorris et al. 2005). The stages are (1) forming: how the group forms, how members are orientated to the group, the establishing of ground rules and orientation to the task; (2) storming: a period of conflict, polarisation and emotional responses to the task; (3) norming: group cohesion and norms are established with an emphasis at finding solutions; (4) performing: the group becomes flexible, functional and adaptable and achieves the task required; and (5) adjourning: the group is dissolved (Tuckman and Jensen 1977). We did not use the fifth stage of the model as the partnership is still in existence. We did not conduct specific analysis of programme data using the Tuckman model but use to frame our experience. We use findings and quotations from our PRIME consortium satisfaction survey (Box 1) and indicators from our logframe and theory of change (Fig. 1) within the stages of Tuckman’s model to support our discussion.

## Forming

In 2007, the Lancet Global Mental Health Group issued a call to action (Lancet Global Mental Health Group et al. 2007) to governments, donors, multilateral agencies and other stakeholders to scale up services for mental health globally. As part of this, they suggested that research is needed to determine whether mental healthcare can be delivered by non-specialist healthcare workers and how this could be provided in routine healthcare settings within the health system. In the years that followed, there has been a burgeoning of new global mental health research funding from agencies including the US National Institutes of Health, Grand Challenges Canada, the European Commission and the Wellcome Trust. In 2010, DFID advertised a call for applications for funding for a 6-year-long Research Programme Consortium for Improving Mental Health Services in Low and Middle-Income Countries. This was awarded to the Programme for Improving Mental Health Care (PRIME) (Lund et al. 2012).

Initially, the bid was led by the late Alan J. Flisher of the University of Cape Town, a South African psychiatrist, psychologist and global mental health researcher who was a strong advocate for developing mental health policy and programmes in Africa. He led the Mental Health and Poverty Project (MHaPP), DFID’s initial mental health bid which ran from 2005 until 2010 (Flisher et al. 2007).

**Fig. 2:**
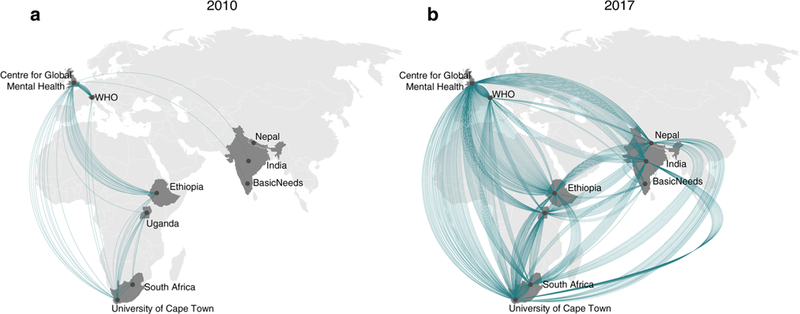
Cumulative PRIME partner collaborations on peer-reviewed publications before the partnership (up to 2010) and at the end of six years (up to 2017). Each line represents a collaboration between two partner organisations on a single peer-reviewed publication. If a paper had multiple co-authors at different partner organisations, there will be lines between each of the partner organisations

The partners included within the bid were Addis Ababa University, Ethiopia; Sangath and Public Health Foundation of India, India; Transcultural Psychosocial Organisation (TPO), Nepal; University of KwaZulu-Natal, South Africa, Makerere University, Uganda, the Centre for Global Mental Health (King’s College London and London School of Hygiene and Tropical Medicine), United Kingdom; BasicNeeds, India; and the World Health Organisation (WHO). Some of the partners had existing collaborations whereas others were new partnerships. These existing relationships can be seen in Fig. 2 which describes the collaborations on academic publications prior to 2010.

The academic partners in PRIME implementation countries were country-based and chosen because of their experience in global mental health research. A Ministry of Health partner in each PRIME implementation country was an integral part of the partnership and participated in the development of the proposal. When Flisher died prior to the submission of the proposal in 2010, the leadership was passed on to Crick Lund, another global mental health researcher at the University of Cape Town. Thus, the leadership remained with the LMIC institution. Crick Lund became the chief executive officer (CEO) and Vikram Patel and Mark Tomlinson were the research directors who were also based in LMIC countries (India and South Africa respectively). Therefore, the LMIC leadership of the agenda and operation of the partnership was a guiding principle even before the start of PRIME.

PRIME commenced in May 2011. The programme was divided into three main phases: (1) the inception phase where we conducted formative work to develop context-specific integrated mental healthcare plans for one district or sub-district in each of the PRIME countries, (2) the implementation phase where we implemented and evaluated these mental healthcare plans in each district; and (3) the scaling up phase where we scaled up these mental healthcare plans to other areas within the district or neighbouring districts. This is described in detail in Lund et al. (2012).

A key part of the inception phase was used to set up detailed Terms of Reference for each partner, establish governance groups (Table 1) and develop strategies and policies (Table 2). All these policies and strategies were set up with the following partnership principles in mind, based on the experience in MHaPP (Mirzoev et al. 2012): (1) mutual respect and listening carefully to partners’ views and priorities; (2) fairness and transparency in the allocation of resources based on allocated work; (3) clear roles and (high) expectations of each partner; (4) flexible and flatter management structures to promote equity and trust between partners; (5) regular, open channels of communication; and (6) building long-standing relationships built on openness and trust.

## Storming

PRIME undertook an ambitious task: to provide research evidence for the development, implementation and scale up of district-specific mental healthcare plans using the same framework in five countries (Lund et al. 2012). Each country had different cultural contexts, types of preliminary research available, research capacity, health systems resources, policy environments and expectations from Ministries of Health. We needed to ensure a balance between a generalizable approach to the implementation and evaluation of our mental healthcare plans while being mindful of the contextual differences and challenges in each of the PRIME countries. PRIME partners came from a variety of disciplines and included psychologists, psychiatrists, epidemiologists, programme managers, economists, medical doctors, researchers and policy makers, each with their own rationale for joining the partnership. Some LMIC partners had also previously been involved in inequitable partnerships with HIC where there was a feeling that LMIC partners were seen as field researchers rather than equal members. As a result, building trust between partners was considered a critical step in order to work effectively.

Given the complexity, diversity and history of the programme, PRIME’s storming phase occurred primarily during the inception phase and the beginning of the implementation phase, when detailed planning for the remainder of the research programme was required. At this point, a large number of methodological decisions needed to be taken across the partnership. Some of these questions included the following: what were the key cross-country research questions about the feasibility, acceptability and affordability of integrated mental healthcare plans? What should be the core components of the mental healthcare plans implemented in each country? To what extent could these components vary across countries? How should we balance the priorities of Ministry of Health partners across countries? What are the ways to evaluate the implementation of the PRIME mental healthcare plans using a common evaluation framework across all five countries? How can the studies be carried out with scientific rigour but within the financial and human resource constraints of the programme? These were complex questions requiring input from all partners in the consortium.

A challenge during this period was establishing the relationships between cross-country partners based primarily in HIC and country partners (all based in LMIC) in terms of the management of the partnership, the input into the research designs and decisions about implementation. In the initial proposal, the research evaluation component of PRIME was to be led by the Centre for Global Mental Health in London. However, it became clear early on that the development of the integrated mental healthcare plans would need to be context-specific. In addition, principal investigators from each country were best placed to consider the existing level of evidence in each country, methodology and design of the research, feasibility issues around the collection of data, specific research tools which had been validated in their country and the capacity (and capacity building needs) of their implementers and researchers. As one partner remarked in the consortium satisfaction survey (see Box 1 for an outline of the methods), “In general there is a potential for disconnect between country PIs and cross-country partners. Periodically this becomes problematic. In retrospect it would have been better to have involved country PIs earlier in developing methodology. Perhaps even better would be to have country PIs on the methodology development team (although the consortium started like that, the discussions tended to start with cross-country PIs and become quite advanced before country PIs were involved). The issue is accentuated by communications difficulties for country partners (e.g. slow internet, difficulties connecting to teleconferences).” This led to some tension within the partnership, with country principal investigators feeling that they were not sufficiently involved in the decision-making process. As another partner remarked in the consortium satisfaction survey, “research designs were developed a bit top-down (cross-country to country), especially in the beginning of the program”. Partners remarked that this could be improved by “encourage[ing] country partners to actively participate in the cross-country management and planning of the project”.

A major shift in the research planning happened during annual meeting in April 2012 where the draft version of evaluation protocol was significantly revised through discussion with country principal investigators and cross-country partners. This included the addition of protocols developed by LMIC partners. For example, there was initially no plan in the cross-country protocol to conduct a community survey. However, the Ethiopian team presented the results of their community survey which was conducted in the first grant year to determine the prevalence and treatment of depression and alcohol use disorders. As this was a key knowledge gap in the PRIME countries, it was decided by agreement between all partners to include this in the cross-country protocol.

Following this, the partnership changed its approach to evaluation design development to ensure that country principal investigators were involved actively at each stage of the process to ensure more efficient and contextually appropriate evaluation design. This was facilitated by additional monthly meetings and by co-ordinating working groups for the development of specific protocols. These groups were co-ordinated by a researcher from the Centre for Global Mental Health in London, mentored by a senior member of the partnership (usually the CEO or research director) and included all country principal investigators. One partner remarked in the partnership consortium satisfaction survey (Box 1) that “things have improved a lot since we have had more specific working groups with a lead person for each design”. Decisions were made by consensus although, at times, this required further discussion with the PRIME leadership before it was agreed upon. For example, in South Africa, an exemption from the cross-country protocol was requested. The partner put the case that to conduct a community-based survey to assess population-level changes in district level mental health treatment coverage made little sense because the South Africa Department of Health identified chronic disease patients as a priority group for mental health service provision in primary health care facilities. The implication of this strategy was discussed in detail with the research directors and CEO who had to decide how to ensure cross country comparability while ensuring contextual relevance. Ultimately, the decision was taken not to conduct the community survey in South Africa. Instead, additional funding was obtained to conduct a pragmatic cluster randomised control trial to provide evidence of the effectiveness of the scale up of the facility intervention to the South African Department of Health. There have been many such discussions throughout the course of PRIME with the research directors and CEO to determine to what extent country partners could adapt or change cross country methods to suit their contexts and interests.

There were various ways in which we ensured learning and exchange between countries. These included our monthly principal investigator teleconferences with a standing item of country updates of progress and challenges, our annual face-to-face meetings where each country presented their findings, sharing of draft MHCPs, training manuals, all published papers and the forming of informal networks and friendships across countries.

An often controversial issue for academics is data ownership and publications. Although data ownership and access to data was explicitly mentioned in the sub-contracts with all the partner organisations, we proactively developed a publication policy (http://bit.ly/2BwiZu2) for the partnership on how data would be shared for publication. This was based on the Aspen/ Indigo network (Lasalvia et al. 2013) publication policy (Table 2). The policy explicitly encouraged publication by junior researchers and active collaboration between PRIME partners and with external collaborators. In addition, the policy outlines that papers should include a mix of cross-country and country authors and that papers based on country findings should generally be published before cross-country papers, or at least there should be consultation to ensure that country papers would not be disadvantaged. The policy included an Intention to Publish Form to be completed by the lead author which outlined the title, authors, abstract, types of data, target journal and submission date for proposed papers and was circulated to the partners via the PRIME Management Team. Where there were overlaps between planned papers, a discussion to find a solution was moderated by the CEO. Although this system functioned relatively well, some PRIME partners stated at our second annual meeting that this system was affected by the assertiveness of individuals and was too ad hoc to ensure that key partners were acknowledged for the intellectual contribution to PRIME. As a result, a publication list was created with the potential outputs of PRIME, both inter- and intra-country. This was circulated to the PRIME partners who nominated papers which they would like to lead, co-author or mentor. To ensure that potential high impact papers were allocated equitably, partners nominated papers which they wanted to lead. These were allocated fairly across the partners. In some cases, where there was more than one partner who wanted to lead the paper, this was negotiated by joint discussions between the authors and CEO. The publication policy was also revised regularly to incorporate discussions about access to and ownership of data and the composition of co-authors from country teams and cross-country teams.

## Norming

By the end of the second grant year we entered a “norming” stage of the partnership where the partnership and lines of accountability were established, administrative and management procedures were in place and capacity was developed (where necessary) to undertake specific tasks. The formative research and piloting had been completed and baseline studies in the implementation phase had started in some countries.

During this period, we conducted the consortium satisfaction survey (Box 1). When asked what things were good about the partnership and what could be improved, many of the partners mentioned the principles on which the partnerships were based on the following:

1. Mutual respect and listening carefully to partner’s priorities: partners reported that there was “mutual respect” with “appropriate attention and value... given to even a small suggestion and comment”.
2. Fairness and transparency in the allocation of resources based on allocated work: the partnership was seen as “open and transparent in decision making for research, capacity building and resources”.
3. Clear roles and (high) expectations of each partner: partners noted the “wealth of skills to share between members” and “high level of commitment and intellectual capital”. They noted that there should be more attention given to forward planning with “a more realistic approach towards what we set as our goals” and “an understanding of where we are heading in next three years”.
4. Flexible and flatter management structure to promote equity and trust between partners: partners remarked on the “great leadership”, the “program structure with program directors/PIs and country PIs & overall management of program!” However, some partners felt there was scope for more “involvement in decision making on management issues” and a more efficient process for making decisions. As described above, there was particular reference to the “balance between country and cross-country partners in terms of leadership”. The “administrative burden” and “workload management for team members” was seen as an area for improvement.
5. Regular, open channels of communication: the experience of communication within the partnership varied, some felt that “the consortium is open and there is opportunity to raise concerns at a country level”. However, as one partner noted, “The relationship between country and cross-country partners can be difficult given their different roles. It would be important to improve communication as well as realise how the different roles people play are part of the larger whole”. Face-to-face meetings were seen as valuable, the poor technical quality of teleconferences and the focus of the meeting agenda on updates rather than troubleshooting were seen as areas for improvement.
6. Building genuine, long-standing relationships based upon openness and trust: partners commented on “trust and openness amongst members of the consortium” and that they enjoyed “the friendships and collaborations that are being forged which will endure well beyond PRIME”.

More results can be found in Supplementary File 1.

## Performing

The “performing” stage of Tuckman’s model describes how once the group has an established way of working it starts to work towards common goals and performing with a high level of success. We describe our successes and challenges in relation to the four key outputs which we set out in our logframe and theory of change (Fig. 1): management, research, research uptake and capacity building.

### Management

#### Successes

We aimed to establish and maintain an efficient and well managed partnership which drew on the strengths of each partner to deliver a high-quality research programme that achieved the programme’s aim. As described above, we established key governance groups (Table 1), policies and strategies (Table 2) according to our partnership principles. These were revised regularly throughout the programme.

The internal governance groups within the generally worked well with meetings conducted as planned. However, it became apparent early on that more frequent meetings would be necessary to discuss the details of PRIME research. In addition to our regular governance meetings and monthly meetings, we conducted face-to-face meetings with key PRIME members when they were attending other events (amounting to five additional meetings over the first six years of PRIME). Face-to-face meetings, both our annual meetings and additional meetings, were essential to planning the work, building good working relationships and establishing trust between partners.

The functioning of external governance groups varied. In Ethiopia, India and Uganda, the community advisory boards provide oversight to the research and met at least biannually. Members included senior district officials or community leaders, faith leaders and either mental health service users or caregivers of people with a mental illness. In Ethiopia, all key district cabinet offices were represented, in addition to public and faith leaders. This meant that the advisory board could influence the district policy makers in a tangible way. For example, the community advisory board helped drive the decision by the district to make medicines available freely for 1000 people with serious mental illness and the subsequent implementation of this decision. In Nepal, the CAB was established but meetings were not as frequent as in other countries due to frequent transfer of district level government officials. The Nepal CAB did not manage to provide the intended oversight and was therefore discontinued in later years. Input from community and government officials was obtained in other or ways such as a prioritisation exercise of the mental disorders to be included in PRIME (Jordans et al. 2013) and stakeholder meetings at national, district and municipal levels. In South Africa, a separate community advisory board was not constituted. Instead, the South African team leveraged existing clinic advisory boards and district management structures. Although the Consortium Advisory Group met biannually and provided oversight and input in relation to research, capacity building and research uptake, the scope and complexity of PRIME meant it was difficult for the Consortium Advisory Group to give in-depth feedback on the research component of the programme.

An important part of the success of PRIME was financial stability due to the length of the initial 6-year funding period. This stability over an extended time ensured continuity so that we could engage locally and internationally on a sustained basis and to complete a large amount of high-quality work across five countries. We could conduct thorough formative work and spend more time testing our implementation and evaluation methods with less pressure to immediately apply for further funding. It has provided a significant platform for obtaining funding, with the receipt of more than 25 million pounds in other funds to work towards PRIME-related goals.

Apart from the model of funding, PRIME benefited from how DFID managed their role as a funder. DFID allowed for flexibility within programme as long as we met the main aims and objectives of the research programme. DFID measured progress by the extent to which we fulfilled our logframe targets in relation to management, research and research uptake. Their particular focus was on the impact of the research and to what extent our research was taken up in policy and practice in study countries and internationally. DFID did not get involved in the details of the research project, for example the decisions about the research design of the project, ethical or regulatory approvals of the programme, the development of the mental healthcare plans or the evaluation of the data apart from their role in the Consortium Advisory Group. This flexibility from DFID allowed us to broaden the scope of work to ensure that the research could be contextually relevant. The relative autonomy we were given ensured a high level of motivation and group coherence amongst PRIME partners and allowed us to be more productive than we initially expected. For example, our logframe target for the end of the six years was initially 20 papers in peer-reviewed journals. This was increased to 40 and eventually 65 papers in peer-reviewed journals. By the end of year 6 we had published 67 papers in peer-reviewed journals. This is in contrast to other funding mechanisms where the research processes and content of the programmes are highly regulated and may not allow adaptation across contexts.

Because of these successes, PRIME achieved an annual rating of at least A by DFID each year, which indicated we were meeting or exceeding their expectations. DFID also invited us to apply for two years of additional funding. This funding was granted on the basis or our performance and is being used to consolidate our existing work and ensure that our findings are translated into policy and practice.

PRIME departs in several ways from a traditional model of research partnerships in global health where HIC countries make decisions and lead research with LMIC partners implementing the research. The leadership of the consortium was based in middle-income countries (MIC), India and South Africa. This has meant that the leadership are well-acquainted with the difficulties of working in resource constrained settings and were actively involved in mental health policy and planning in their respective countries. However, the resources and institutional capacity which could be harnessed from the University of Cape Town was likely higher than if the RPC was led from a low-income country (LIC). This includes support for financing, contracts, data storage, access to software and libraries. In addition, there is a fluidity amongst PRIME individuals in relation to their identities, experiences and HIC or LMIC institutional affiliations. Many individuals who were formally affiliated with high-income institutions have spent a proportion of their personal and professional lives working and living in LIC and therefore have an in-depth understanding of the health system and culture of those settings. For example, Vikram Patel (PRIME Research Director) was affiliated with both the London School of Hygiene and Tropical Medicine and Sangath, India. In addition, Charlotte Hanlon, PRIME researcher and later Research Director, was employed by King’s College London and Addis Ababa University and lives and works in Ethiopia. This was possible because the London School of Hygiene and Tropical Medicine and King’s College London have allowed their faculty to be physically based outside of London, thus allowing them to spend a considerable amount of their time living and working in LMIC with access to the mentorship, resources and prestige provided by a HIC institution. The flexibility of these UK institutions have contributed to PRIME’s success.

#### Challenges

As mentioned above, the establishment of trusting relationships between partners, especially country and cross-country partners needed time at the start of the programme. However, this was resolved during the initial stages of the programme to engage in open and transparent discussions. Productive working relationships became the norm between all partners.

A major challenge for the implementation country partners was the financing mechanism, a spend-and-claim model, where spending was reimbursed on a quarterly basis. This resulted in a five-month gap from the start of spending to reimbursement. This was particularly challenging for low-income country universities and small NGOs who did not have adequate reserves to fund the programme in advance, resulting in some programme delays.

### Research

#### Successes

During the six years of PRIME, we aimed to produce a body of policy relevant research which could help understand the implementation and scaling up of packages of care for mental disorders in low resource settings. We did this by developing and implementing district-specific mental healthcare plans in all five countries (Fekadu et al. 2016; Jordans et al. 2016; Petersen et al. 2016; Shidhaye et al. 2016) (Hanlon et al. 2016; Kigozi et al. 2016) and scaling them up in Ethiopia, India, Nepal and South Africa.

**Fig. 3:**
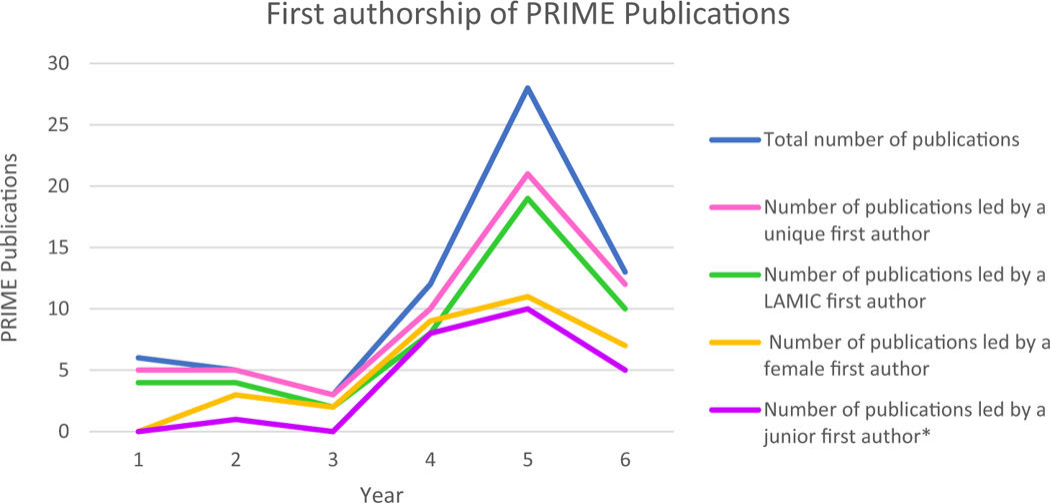
First authorship of PRIME Publications during the first six years of the grant (1 May 2011–30 April 2017). The asterisk indicates junior authors defined as those who have no PhD or graduated less than five years ago and are not a principal investigator on PRIME

**Fig. 4:**
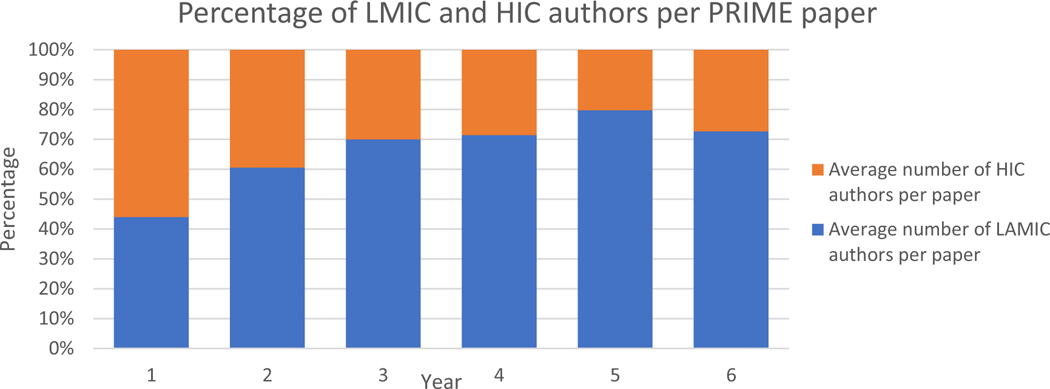
Average percentage of authors per publication by HIC and LMIC during the first six years of the grant (1 May 2011–30 April 2017)

We have made 63 presentations at international conferences and published 67 peer-reviewed publications. Of these, 47 of the first authors were based in LMIC reflecting the considerable intellectual contribution that has come from LMIC partners (Fig. 3) with the proportion of LMIC authors increasing over time (Fig. 4). Most papers involved collaboration across partnerships. By the end of the initial six years of PRIME, all nine PRIME partners had collaborated on at least one peer-reviewed publication using PRIME resources or data with most collaborating more frequently. Figure 1 presents the change in collaborations on all peer-reviewed publications, demonstrating that PRIME has substantially increased joint collaboration between partners.

There are several likely reasons for this success. Working in partnership allowed us to draw on skills across the consortium and develop common research questions and study designs which could be adapted across countries. It also provided a platform for shared learning which was particularly helpful in the development of the district mental health care plans and the sharing of interventions developed by the different countries. Because of the expertise within the PRIME partnership, the research was conducted to a high standard with mentorship of midlevel and junior researchers by senior researchers throughout the research process. The number of research outputs also indicate the extent of PRIME research: there were multiple study designs to answer multiple research questions across all five countries in all the three phases of the project. These research questions often went beyond the outputs required by the funder but were conducted because they answered questions important to global mental health. This indicates the high level of personal commitment of PRIME partners to the field and has resulted in a more ambitious and extensive research programme.

#### Challenges

A significant challenge has been completing the research programme in the allocated time frame. We faced delays early in the programme for several reasons: (1) contextual factors, such as difficulty mobilising financial resources from the Ministries of Health for the implementation of the mental healthcare plans and the identification of appropriate cadres of health care workers; (2) our ambitious programme of research; and (3) the complexity of the programme.

The complexity of PRIME changed in two ways since the funding was granted. The first was the complexity of the mental healthcare plan. During our formative research, it became apparent that a more comprehensive mental healthcare plan was needed than originally planned. This extended the formative phase and resulted in more complex evaluation designs. For example, we added pre- and post-community and facility surveys to measure the change in treatment coverage and facility level detection of depression and alcohol use disorders as a result of PRIME. Because of the number of key partners and the contextual variations between countries, a relatively large amount of time was taken to design a protocol that all partners agreed on. As mentioned above, during the latter half of the programme, this process was made more efficient by creating small groups responsible for each evaluation design. Despite the delay, we believe it led to the development of practical, contextually relevant mental healthcare plans and rigorous evaluation methods which resulted in more applicable research findings. This was possible because PRIME was a well-functioning consortium.

### Research Uptake

#### Successes

Our Research Uptake Strategy was informed by the Overseas Development Institute’s Research and Policy in Development framework (Hovland 2005) and DFID guidelines on research uptake (Department for International Development 2005). A stakeholder analysis (Makan et al. 2015) ensured that our research was tailored to stakeholders in each PRIME implementation country. We identified four key audiences for our research: (1) researchers and health practitioners; (2) people affected by mental illness, their families and the broader community; (3) civil society and the media; and (4) policy makers and donors.

As a partnership we aimed to effectively communicate our research findings and ensure research uptake by national and international stakeholders to influence policy and practice, both in the study countries and other LMICs. We conducted 224 meetings with district, state and national policy makers over the course of PRIME. We disseminated more than 80 PRIME information products to policy makers and donors, including policy briefs, website articles, research tools, newsletters, posters, brochures and infographics. Most of these products were developed by our full-time research uptake officer. These information products were disseminated at high-level events, for example the “Out of the Shadows” World Bank event in Washington, DC, in 2016, and at a special sitting of the South African parliament’s Portfolio Committee on Health on World Mental Health Day in 2014. In 2016, we published a ten paper supplement in The British Journal of Psychiatry (Breuer et al. 2016; Chisholm et al. 2016; De Silva et al. 2016; Fekadu et al. 2016; Hanlon et al. 2016; Jordans et al. 2016; Kigozi et al. 2016; Lund et al. 2016; Petersen et al. 2016; Shidhaye et al. 2016) which was launched at the WHO mhGAP Forum meeting in October 2015.

As a result of this, the PRIME consortium and PRIME research has been cited 177 times in the media and in 10 international documents which are likely to contribute to development goals by international development agencies. We have also been invited to be involved in policy development in all PRIME countries. Specifically, the revision of the Ethiopian National Mental Health Strategy (Federal Democratic Republic of Ethiopia 2012), New Pathways, New Hope: National Mental Health Policy of India (Government of India 2014) and the Development of the State Mental Health Action Plan, Madhya Pradesh, a template for a community mental healthcare package in Nepal (Government of Nepal 2017), the National Mental Policy Framework and Strategic plan (2013–2020) in South Africa (Republic of South Africa 2013) and the revision of the National Mental Health Strategic Plan (2013–2018) in Uganda. Internationally, we have contributed to various documents such as “Mental Health for Sustainable Development. A report from the All Party Parliamentary Group on Global Health and the All Party Parliamentary Group on Mental Health” (De Silva et al. 2014). Seven of our PRIME partners are commissioners on the Lancet Commission on Global Mental Health and Sustainable Development, which will be published in late 2018.

We have evidence that we reached researchers and practitioners. We have had 1606 citations of PRIME research in academic publications (Google scholar April 2017) and have examples of influence on guidelines or training in all five countries: (1) In Ethiopia, PRIME lessons were used to inform the Ethiopian adaptation and roll-out of the mhGAP action programme; (2) in India, the PRIME training was adapted to provide training for medical officers and establish mental health treatment services through Mann-Kaksha in all 51 districts in Madya Pradesh as part of the state-wide Scaling Up Opportunities for Healthy and Active Minds initiative; (3) in Nepal, we worked with the National Health Training Centre to ensure our training manuals were developed and adopted; (4) in South Africa, we specifically strengthened and revised the mental health and substance use components of the Adult Primary Care guidelines (Republic of South Africa 2017) which are being used nationally; and (5) in Uganda, PRIME played a pivotal role in the adaptation of the mhGAP Intervention Guide and the subsequent training.

The success of PRIME in relation to getting research into policy and practice is likely to be a result of strong relationships and buy-in from the Ministry of Health. We achieved this in several ways. First, we aligned the PRIME programme with the agenda of the Ministries of Health by the including Ministry of Health partners in PRIME from the grant proposal stage. For example, in South Africa, we chose the implementation district at the request of the National Department of Health because it was a pilot site for Integrated Clinical Services Management and the new National Health Insurance. Second, we worked with facility, district, provincial/state and national level stakeholders in the development of the PRIME mental healthcare plans using Theory of Change Workshops (Breuer et al. 2014). This meant that the mental healthcare plans were tailored for routine settings and could be integrated into routine health services using mostly existing staff. This also increased the probability that the changes would be sustained after PRIME. Third, in several instances our work in PRIME was a continuation of a long-standing relationship with the Ministries of Health. For example, in Uganda, partners had a good working relationship with Ministry of Health partners which had been strengthened during the Mental Health and Poverty project. This relationship has continued and strengthened through the work of PRIME. Instead of taking a critical and antagonistic stance towards governments in PRIME countries we have tried to foster a relationship of mutual benefit where PRIME partners have become trusted experts which the Ministry of Health can rely on to provide evidence and to help develop policies for mental health service provision and systems strengthening. Fourth, although policy makers have not attended PRIME consortium meetings after the first few years, we have prioritised ongoing relationships with policy makers over time and have kept them informed of progress as the project as progressed.

Since the start of PRIME there has been some evidence for a governmental budget increase for mental health in Ethiopia, India and Nepal with mixed evidence from South Africa and Uganda. In Ethiopia, this includes an increase in the procurement of psychotropic medications, training for mental health professionals and the planned scaling up of mental health services in over 200 health facilities and the associated costs in training and supervision (Wolde-Giorgis 2017; The Federal Democratic Republic of Ethiopia Ministry of Health 2015). In Madhya Pradesh, India, there was a 35% increase in mental health budget from 2015–2016 (32.9 million rupees) to 2016–2017 (44.5 million rupees) (National Health Mission 2017). In Nepal, an increase in spending on mental health has been committed to by the Nepal Ministry of Health. This includes six mental health drugs in the free drug list (Primary Healthcare Revitalisation Division 2017) and training of health workers using the PRIME approach which has been endorsed by the National Health Training Centre (National Health Training Centre 2018). In South Africa, there is no current evidence for an increase in the mental health budget but some PRIME partners are working with the National Department of Health to develop an investment case in mental health, drawing on the PRIME model. In Uganda, funding has not changed as a percent of the health budget because the allocation formula is the same. However, there has been an increase in recruitment and training of key mental health personnel in the PRIME implementation district (Kamuli) as a result of PRIME. The above changes across countries can be attributed at least partially to PRIME.

#### Challenges

Despite our successes, the partnerships with Ministries of Health have not been without challenges in all countries. In some countries, such as Ethiopia, Uganda and South Africa, there have been strong ongoing links with Ministry of Health and frequent meetings. In India and Nepal, Ministry of Health involvement has been fragmented at times. There was initially high support for PRIME and a significant amount of involvement but frequent turnover of staff at the Ministries of Health have impacted the involvement of the Ministry of Health partner in some countries. We have mitigated these challenges by engaging a broad range of policy makers at different levels of the health system (district, provincial/state/zonal and national) as well as engaging frequently with Ministry of Health partners. However, strong relationships with policy makers do not always result in changes in practice where no resources are available. For example, in Uganda despite support for PRIME, there are minimal resources for scaling up the services to other geographical areas.

As part of our research uptake strategy, we planned to reach people affected by mental illness and engage them in service planning. This has been a challenge for several reasons. The first is that in some countries such as Ethiopia where no services were available in the district prior to PRIME, most people with mental illness were too unwell to contribute to service planning and there were low levels of baseline mobilisation (Abayneh et al. 2017). In addition, there were no service user organisations active in the PRIME implementation districts at the start of the programme, which meant that any service user engagement would be with an individual rather than an elected representative of a group. However, in each PRIME country, we found ways to include people affected by mental illness. In Ethiopia, Uganda and India people affected by mental illness sat on the community advisory boards. In Uganda, we worked with BasicNeeds to develop service user groups in the district. In Nepal and Ethiopia, people with a lived experience of mental illness assist in the training of service providers.

### Capacity Building

#### Successes

The strength of our capacity building strategy, based on Ghaffar and colleagues’ systems approach to capacity building, was to combine individual training and organisational development and institutional strengthening (Ghaffar, Ijsselmuiden, & Zicker, 2008). This has resulted in flexibility to address the capacity building needs of each partner organisation and individuals within that organisation. For example, we only conducted formal capacity building activities at the initial three annual meetings, opting instead for informal writing workshops at annual meetings which comprised of small group meetings of co-authors in relation to planning, analysis and writing of PRIME outputs. These groups included junior, midlevel and senior researchers which allowed for mentorship in the research and publication process. Additional capacity building activities included attending local courses, for example leadership in global mental health, and data analysis. This allowed each partner organisation to ensure that their specific capacity building needs were met and context-specific capacity building opportunities were realised. It has also reinforced the principles of the partnership that there are no universal “experts” but rather people with expertise in specific areas.

By the end of the initial six years of funding, PRIME had a cumulative total of 20 PhD students (14 women, 16 LMIC-based). By the end of the original grant period, two had graduated and four submitted their dissertations. These researchers have been supported to conduct their PhDs by PRIME and have been mentored in the conduct and write up of their work. Although 8/20 students were part of the Mental Health Epidemiology at Addis Ababa University, others were distributed across PRIME partner organisations including University of Cape Town, the University of KwaZulu-Natal in South Africa, the London School of Hygiene and Tropical Medicine and the Institute of Psychiatry in the UK. Some students also registered at other universities and conducted their research within PRIME. The supervision arrangements were often collaborative: seven PhD students were formally supervised by supervisors in more than one partner organisation. Other PRIME partners provided mentoring and hosted PhD students for research visits. Funding for the PhDs came from various sources including PRIME (particularly for the fieldwork), scholarships such as the Wellcome Trust, self-funding from the students and employment: eight of the PhD students were employed to perform other roles within PRIME such as principal investigator, project, data or site management. Although this has led to these students gaining research experience in areas of their project wide than their PhD project, there have been some delays in their graduation due to these other responsibilities.

The focus on capacity building is evident in our publications by the initial grant period shown in Fig. 3. Not only did the number of publications of the consortium increase over time, the authorship of the papers was distributed across the members of the consortium as indicated by the number of unique first authors. Many of these papers were led by junior authors. Of the 67 peer-reviewed journal articles, 24 were led by junior authors (Fig. 3) with 19 of these by PRIME PhD students. Seventy percent of PRIME publications have been led by partners in LMIC with the percentage of LMIC authors per publication increasing over time (Fig. 4).

In order to embed capacity building in institutions and build sustainable capacity, we supported formal postgraduate programmes, specifically the MPhil in Public Mental Health at the Centre for Public Mental Health in Cape Town (45 students registered from 2012 to 2017) and the MSc in Global Mental Health at the Centre for Global Mental Health in London (145 students registered from 2012 to 2017). PRIME partners also support the PhD programme in Mental Health Epidemiology at Addis Ababa University, which had 20 students registered at the end of April 2018.

We also aimed to build sustainable collaboration within the PRIME partnerships. There is evidence that we have achieved this. PRIME partners have collaborated with each other on other projects and the resultant publications. Key examples of this include the development and production of a mental health volume of the 3rd edition of Disease Control Priorities (DCP 3) (Patel et al. 2016); the Emerald EU FP-7-funded grant investigating mental health systems strengthening (Semrau et al. 2015); a Grand Challenges Canada-funded research programme (mhBeF) implemented in Liberia, Uganda and Nepal (Jordans et al. 2017); and a Global Health Research Unit on Health System Strengthening in sub-Saharan Africa funded by the UK’s National Institute for Health Research (www.healthasset.org).

The majority of our capacity building within the health system occurred as part of the mental healthcare plan implementation. This included training of community level workers such as health extension workers (Ethiopia), village health teams (Uganda) and female community health volunteers (Nepal). We trained facility level service providers such as medical officers and nurses as well as specialist providers. We also conducted training at district level, for example in health information systems, financial management and change management.

#### Challenges

Although we included capacity building for our national or state Ministry of Health partners in our capacity building strategy, the extent to which this was implemented was limited. This is likely due to the priorities of Ministry of Health officials which seldom extend to capacity building in the traditional sense of the word. Instead, we provided specific information and resources relevant to developing or implementing policy. The high turnover of Ministry of Health staff in some countries made it difficult to engage with, determine and respond to capacity building needs. Capacity building for policy makers was explored further by our partner programme, Emerald, which focused on health systems strengthening for mental health (Semrau et al. 2017).

Another challenge we faced were the entrenched historical divisions of gender and ethnicities within all the countries where PRIME partners were based and globally. We were aware of these and tried to address these in how we recruited staff, formed the partnerships, shared the leadership roles and assigned authorship. A limitation of PRIME is that we did not explicitly develop a policy to address these divisions within our partnership beyond the institutional practices of some partner organisations. Examples of these institutional practices include specific aims to increase racial and gender diversity in at the University of Cape Town and the University of KwaZulu-Natal in South Africa through employment equity requirements to redress historical disadvantage. King’s College London and the London School of Hygiene and Tropical Medicine have signed up to initiatives such as Athena Swan which aims to decrease gender disparities in academic institutions in the UK. Despite no formal approach to gender within the consortium we have had, PRIME enabled the first two women to join the PhD programme in mental health epidemiology at Addis Ababa University, and we have had cross-country success in relation to capacity building for women as shown by women leading papers and enrolling and graduating with PhDs. Sister programmes like AMARI which only focus on building PhD and post-doctoral capacity may have more scope to address structural barriers (AMARI: African Mental Health Research Initiative 2018).

## Discussion

PRIME has highlighted some of the potential benefits and challenges related to the formation of multi-country collaborative research partnerships in global mental health. We have shown that by working towards a common goal with strong southern leadership and a collegial relationship between partners, a large and complex research project can be successful. Despite this, the initial stages of the consortium required time to build trust and create a safe space to express different views. This process facilitated implementation country partners to have an influential voice in the decision making within the partnership. In that way, it was possible to balance the contextual environments with the quality and appropriateness of the research. The partnership went on to perform successfully based on objective indicators from our logframe and theory of change in relation to management, research, research up-take and capacity building. Qualitative evidence from our consortium satisfaction survey indicates we upheld many of our partnership principles although partners mentioned several areas for improvement. In Box 2, we have distilled seven of the key lessons which we have learned through PRIME and would use in future global (mental) health partnerships.

Although it is tempting to attribute the success of the partnership to these key lessons, it is worth reflecting on the broader environment in which this partnership took place. Chiefly, PRIME partners had galvanised around a common goal. In 2011, at the start of the PRIME consortium there was considerable momentum in relation to the call to scale up mental healthcare in LMIC (Patel and Prince 2010). PRIME was a direct response to the call for action to scale up the coverage for mental health services to give research evidence of how this could be done systematically. Several PRIME partners were involved in this call to action which underpins the level of expertise of the consortium. It shows that there was a shared understanding amongst partners that mental health was a neglected issue in global health, especially in LMICs, and a determination to make a change to this situation. This was combined with a high level of personal commitment to the field. The strong LMIC leadership, with the CEO, both research directors and experienced principal investigators all based in LMIC, ensured that the research was feasible, appropriate and policy relevant.

This all took place amongst growing global awareness of the importance of mental health. For example, in 2013, all UN member states signed the WHO Global Mental Health Action Plan (2013–2020) (Saxena et al. 2013). This momentum underscored the need for policy relevant research to be ready and accessible to policy makers.

This global momentum was mirrored in the field of mental health research, as shown in other funded initiatives, for example the National Institutes of Mental Health Regional Hubs and the Grand Challenges Canada initiatives. This sense of being part of a global movement was an additional support to PRIME partners. It also made it possible to collaborate on other projects and create sustainable working partnerships which are likely to continue beyond PRIME. It may also be that in this time of (relatively) increased funding in Global Mental Health, there has not been a need for PRIME partners to compete against each other for scarce research funding.

In conclusion, we have shown that a well-functioning partnership in global mental health can be built by ensuring underlying principles of equity, fairness and transparency are upheld when working towards a common goal. This requires time and effort to ensure that partners’ needs and priorities are understood and the contexts of each setting is considered. Strong leadership, based in LMIC where possible, and clear management structures which are consistently and fairly applied throughout the life of the partnership are important, particularly in the storming phase of group development. Funders should aim to provide long term funding which allows partnerships to innovate and shift their research priorities towards key questions in their context.

## Supplementary Material

Supplemental File

## Figures and Tables

**Table 1:** Governance groups in PRIME

Name of governance group	Main function	Frequency of meetings	Chairperson	Members

PRIME Management Group	The decision making body of PRIME and ensuring the achievement of objectives	Quarterly with at least one face-to-face meeting per year	Chief Executive Officer	PRIME Chief Executive Officer (CL), Research Directors (VP, MT), Country Principal Investigators and Ministry of Health partners, 1 representative from each partner. Research Uptake Officer, Programme Manager
PRIME Management Team	Overall leadership and responsibility for the day to day management of the PRIME	Weekly	Chief Executive Officer	PRIME Chief Executive Officer (CL), Research Directors (VP, MT), Research Uptake Officer, Programme Manager
Consortium Advisory Group	Provided external guidance to the programme and reviewed programme of work and progress	Twice a year	Independent external	Ten inter-disciplinary senior research and policy experts, at least one from each participating country, who are independent of PRIME, funders and the PRIME Chief executive Officer (CL), Research directors (VP, MT), Research Uptake Officer, Programme Manager
Country Management Groups	Overall responsibility for the implementation of the programme in each country	Every 1–4 weeks	Country Principal Investigator	Country Principle Investigator(s), Ministry of Health partner. Country Project Manager
Community Advisory Board	Guidance and independent monitoring of the implementation of the programme in the (sub)district	2–3 times per year	Independent external	Composition varies in each country but generally senior district Officials or community leaders, religious/faith leaders, traditional healers, people with lived experience of mental illness and/or their carers

**Table 2:** PRIME policies and strategies

PRIME policies and strategies	Purpose	Developed by

Monitoring and evaluation framework	To measure the progress of the partnership against our stated impact, outcome and four outputs: management, research, research uptake and capacity building in the form of a logframe and theory of change (Fig. 1)	PRIME Management Team (PMT) with support from Centre for Global Mental Health and input from all partners
Research uptake strategy	To outline a strategy for systematically increasing the uptake of PRIME’s research in policy and practice by (1) increasing awareness amongst researchers and health practitioners about the impact of mental illness, and how this can be addressed by improving access to evidence-based mental health care; (2) to mobilise people affected by mental illness, their families and key community stakeholders to use PRIME research to advocate for scaling up evidence-based care for mental disorders; (3) to increase the public awareness and engagement with the research findings amongst civil society and the media, including policy champions; and (4) to guide policy makers and donors to utilise research, in particular the PRIME outputs, to scale up using evidence-based mental health systems, integrating mental health into routine primary and maternal health care	PMT based on a stakeholder analysis from all partners; input from all partners
Capacity building strategy	To outline PRIME’s capacity building approach which aims to build sustainable capacity for health research and evidence-informed policy and planning at individual, organisational and system levels. Specifically, (1) to establish each partner organisation as leaders in mental health services research which will continue beyond the life of PRIME; (2) establish collaborative teaching programmes and jointly apply for further research grants; (3) to establish a broad-based, sustained, collaboration between the PRIME partners; and (4) to ensure that each institution will be able to better support high-quality research, independently secure research funding in competition with northern institutions, establish resources for national and regional capacity building and contribute to the needs of other partner institutions	PMT with Ritsuko Kakuma (now Centre for Global Mental Health, London) based on a capacity building needs and skills assessment at individual, organisational institutional and Ministry of Health levels from all partners; input from all partners
Publication policy	To make explicit PRIME’s approach to data storage, data access and sharing, and publication procedures during the life of PRIME by providing (1) a fair and transparent process for publishing outputs from PRIME; (2) ensuring the timely production of high-quality research outputs, (3) building capacity of junior researchers; and (4) collaborating and sharing data to ensure the maximum impact of the PRIME’s work. This includes a transparent intention to publish process where lead authors specified the paper they wanted to publish in collaboration with co-authors, the research question, data analysis approach and target journal	PMT based on the Aspen/Indigo (Lasalvia et al. 2013) publication policy and incorporating DFID Research Open and Enhanced Access Policy v1.1 (Department for International Development 2012)
Expression of interest policy	To provide a clear process for assessing the potential collaborations of parties interested in PRIME through a centralised application process linked to our website and administered by the PRIME Management Team	PMT with input from all partners

## Box 1 PRIME consortium satisfaction survey

In February and March 2014, around midway through the programme, we conducted an internal consortium satisfaction survey. The survey questionnaire was adapted from the RESYSYT satisfaction survey (www.resyst.lshtm.ac.uk). It was circulated to our PRIME partnership mailing list which was sent to all key PRIME partners including PRIME management, principal investigators, policy makers, project-coordinators and other cross country partners. Sixty-four percent (28/44) of PRIME partners participated in the survey. We made the survey anonymous to create a safe space for critical feedback. However, as it was anonymous, we cannot confirm who participated or the reasons for non-participation.

Fifty percent of respondents were based in LAMIC with 5/8 principal investigators and 1/5 Ministry of Health partners responding. We conducted a thematic analysis of the open comments from the end of the survey which asked PRIME partners what they felt was good or needed improvement in the partnership and used these to support the findings of this paper. The results should be interpreted with caution as the response rate was low and it is likely that those who did not participate in the survey are also less involved in the programme and may be less satisfied with the consortium than partners who responded.

The full results of this analysis are available in Supplementary File 1.

## Box 2 Lessons learned from PRIME

1. LMIC researchers should lead global mental health research programmes or at least have equal decision-making power with researchers in HIC (for example as joint principal investigators) for all research conducted in LMIC
2. Operating procedures and decision-making processes of the partnership should be based on the principles of mutual respect, equity, fairness and transparency.
3. All collaborators should be aware of the fluid and the initially challenging process that are normal for group development
4. Partners should allow sufficient time for complex and consultative decision making at the beginning of the programme.
5. Researchers should engage early and frequently with policy makers to build trust and align the programme to the Ministry of Health Agenda to ensuring research is taken up into policy and practice
6. Funders should consider long term grant funding periods (> 5 years) to optimise true collaboration and productivity.
7. Funding in LMIC should be provided up front where possible so as not to exclude LMIC researchers
