# Supplementary material for: Partnerships in a Global Mental Health Research Programme—the Example of PRIME
Source: Glob Soc Welf. Author manuscript; Available in PMC 2020 Jan 24. (PMC6980236; doi:10.1007/s40609-018-0128-6)
Supplement: Supplemental File [file NIHMS1021528-supplement-Supplemental_File.pdf]

Partnerships in a global mental health research programme - the example of PRIME

Global Social Welfare

Table 1: Areas that PRIME partners felt were good in the partnership

Themes		Comments from partners
Partnerships		
	Supportive nature of partnership	*Being part of a like-minded group of people who work in challenging circumstances and can encourage one another*<br>*Members are open to help and support each other*<br>*The mutual respect*<br>*Colleagues' working relationships*<br>*Trust and openness amongst members of the consortium*
	Friendships	*The people and friendships formed*<br>*The friendships and collaborations that are being forged which will endure well beyond PRIME.*<br>*And friendship*
	Opportunity for collaboration and networking	*Nice group to work with*<br>*Open collaboration*<br>*Opportunity to work in a team of researchers*<br>*It gave us excellent opportunity to learn on how to do collaborative work across countries*<br>*Extremely useful research networking opportunities*

		International Collaboration promotes shared learning<br>*Excellent Collegiality*<br>*Cross country Novice and experienced researchers consultation process*<br>*An opportunity for building long working relationship with colleagues from various centres, for future collaboration*<br>*Connecting with people working in the field of global mental health*<br>*High degree of collaboration and respect between partners*
	Expertise of partners	*The range of expertise in the consortium*<br>*Exposure to working with high quality, very experienced researchers*<br>*Wealth of skills to share between members*<br>*High level of commitment and intellectual capital*
	People	*Dedicated people*<br>*Great network or members*<br>*The pleasure of being in such a scientifically stimulating group.*<br>*Stimulating and talented partners*<br>*The people - passionate, brilliant, nice.*
Leadership and management		
	Leadership	*Program structure with program directors/PIs and country PIs & overall management of program!*<br>*Working with UCT. UCT (specifically XXXXX) has been exemplary leader and I have learnt a lot from his*

		leadership, input on research activities and overall support<br>*Great Leadership*
	Management	*Very systematic processes & overall management of program!*
	Opportunity to raise concerns	*Extremely supportive in addressing country-related queries*<br>*Appropriate attention and value is given to even a small suggestion and comment.*
	Decision making	*Open and participatory decision making*<br>*PRIME consortium is open and transparent in decision making for research, capacity building and resources*
	Communications/connectedness	*Frequent face-to-face meeting.*<br>*Networking*<br>*Partners are updated on the consortium progress through quarterly reports and teleconferences.*
Research		
	Understanding of different contexts	*Working with colleagues from different settings provides an opportunity for a better understanding of the contexts in various settings*<br>*Exposure to diverse research and health contexts*
	Flexibility for countries	*Flexibility in development of country specific approaches.*
	Evidence based research	*Evidence based research*
	Scope of work	*Excellent focus on the key questions and issues in the field of GMH at the moment*

		This is the result of working in a complex intervention.<br>*The designers of this study are appreciated*<br>*It gave us a skill to look beyond researching a narrow objective.*<br>*Cutting edge research on implementation and scaling up of mental health care*<br>*The fact that we are answering groundbreaking questions that will have a big impact in the field*<br>*Mental health is strongly advocated as part of health*<br>*The cutting edge health systems science*<br>*Good science*<br>*First hand experience of implementation science*<br>*Exposure to complex research practice, over time*<br>*Interesting research which makes a real world difference*
Research uptake		
	Interaction with experts and policy makers	*Get opportunity to interact with national and international experts/stakeholders*
	Potential impact of work	*PRIME, as a research undertaking, is unusual and very complex. The output has the potential to make substantial contribution to the delivery of mental health care in Ethiopia and other similar settings.*<br>*Opportunities for policy influence*<br>*International collaboration promotes greater possibility of making an impact locally and internationally*

		It has drawn widespread attention and interest and has helped in promoting mental health research *Able to contribute to improve mental health situation in the country* *Collaborations and multiple research opportunities have opened to enhance and develop mental health research and advocacy in country* *Opportunity to develop local models of mental health service delivery*
Capacity Building		
	Capacity Building	*Building capacity on research* *Research capacity development within the Consortium* *Very good capacity building opportunities* *Super capacity building opportunities* *Working with PRIME has provided a great opportunity for building capacity of the project staff.* *Opportunity to work on my PhD as part of the project*
	Mentorship and support for research	*The research consortium receives input from senior researchers that have influenced directly our research group* *Great support from senior team members* *Ability to keep learning*
	Promotes access to additional funding	*International collaboration promotes access of researchers in LAMIC to other grants and funding opportunities*

Table 2: Areas that PRIME partners felt needed improvement

Themes		Comments from partners
Leadership and management		
	Forward planning	*An understanding of where we are heading in next three years*<br>*A more realistic approach towards what we set as our goals*
	Improve relationship between cross country and country partners	*The relationship between country and cross country partners can be difficult given their different roles. It would be important to improve communication as well as realise how the different roles people play are part of the larger whole*<br>*The balance between country and cross-country partners in terms of leadership.*<br>*Research designs were developed a bit top-down (cross-country to country), especially in the beginning of the program.*<br>*Encourage country partners to take lead in crosscountry papers*<br>*Cross country partners from high income countries should spend time in country sites to understand the context and conditions where the research is taking place.*<br>*It would be possible to support some cross-country partners to think in a more rational and realist ways. They could then understand the price of working on a ground and generate data*
	Meetings and quality of calls	*Quality of teleconference*<br>*Improved technology during teleconferences*

		Improved telecommunication systems for monthly/quarterly Meetings<br>*Telecommunications are not always optimal*<br>*Technical quality of the calls*<br>*Telecommunications (although better than it was)*<br>*Meetings are a significant problem, but this comes about because of other linked consortium-based projects In retrospect other models might have worked better for 'adding value' to PRIME*<br>*More leisure time for informal meetings in annual meetings*
	Greater involvement and efficiency in decision making	*Involvement in decision making on management issues.*<br>*More room for consultations at all levels.*<br>*Communication on management issues*<br>*Encourage country partners to actively participate in the cross-country management and planning of the project*<br>*At times, pace of implementation could have been increased I think (in years 1-3).*<br>*Long time to complete certain activities or to take decision on certain aspect e.g. finalizing research protocol, etc etc.*<br>*Decisions around the science of the project need more succinct and important discussion - especially when expected outcomes fail to materialise. I don't know of such a space - annual meetings achieve this to some extent, but a robust discussion of what works and what doesn't work in a meeting of principles may achieve more in providing consensual direction*

	Spend and claim system	*Pay and get refund is a great principle for those who can pay from their pocket. This could be well negotiated with funding agency if it was truly accepted as a problem by every one*<br>*The need to have project money advanced up-front to country teams.*<br>*Payment scheme or delay in payment*
	Workload	*The administrative burden.*<br>*Workload management for team members*
	Budget	*Budget for PI requires substantial improvement compared to the time put in to ensure high quality research output as this is not in tandem with the budgeted time at all.*<br>*More money to pay existing staff for more of their time on PRIME and to pay for more core staff as there is too much work and too few people so sometimes corners are cut which is a shame on a project with this potential.*
	Country to country engagement	*More engagement between the different sites.*
	Nothing	*None*<br>*None*<br>*None*
Research		
	Focus on implementation	*More focus on implementation rather than on evaluation*
	Consistency of evaluation methods	*Improvement required in Capacity building and Research component*

		Development of more consistent evaluation methodologies across country sites.
	Higher prioritisation of country issues	*Country context is more important than cross county issues Countries should be given more leeway to focus on particular issues that are of most import to their country contexts*
	More focus on difficulties of implementation	*More focus on complications and barriers of implementing the mental healthcare plan (e.g. financial resources for scaling up phase).*
	Focus on maternal mental health	*Greater focus and impetus for maternal mental health from consortium leadership, earlier in consortium time line.*
	Greater link with economic development	*Greater links with economic development policy debates.*
Research Uptake		
	Increased Ministry of Health Commitment	*Greater Ministry of Health commitment to scaling up mental health care.*
	Improved research uptake collaboration	*Improved cross-country research uptake collaboration and engagement during the scaling-up phase*
Capacity building		
	More capacity building directed at partners outside consortium	*Capacity building need to also target partners outside the consortium e.g. policy makers, service providers within the implementation site.*

	Communication for PhD students	*More opportunities for communication cross country for PhD students involved in PRIME*
	Proposal writing	*This is perhaps late now, but more input in proposal writing would be helpful*
